# Circulating IL-17F, but not IL-17A, is elevated in severe COVID-19 and leads to an ERK1/2 and p38 MAPK-dependent increase in ICAM-1 cell surface expression and neutrophil adhesion on endothelial cells

**DOI:** 10.3389/fimmu.2024.1452788

**Published:** 2024-10-18

**Authors:** Jérôme Bédard-Matteau, Antoine Soulé, Katelyn Yixiu Liu, Lyvia Fourcade, Douglas D. Fraser, Amin Emad, Simon Rousseau

**Affiliations:** ^1^ The Meakins-Christie Laboratories, Research Institute of the McGill University Heath Centre, Montréal, QC, Canada; ^2^ Department of Medicine, Faculty of Medicine, McGill University, Montréal, QC, Canada; ^3^ Department of Pharmacology and Therapeutics, McGill University, Montréal, QC, Canada; ^4^ Department of Electrical and Computer Engineering, McGill University, Montréal, QC, Canada; ^5^ Children’s Health Research Institute & Lawson Health Research Institute, London, ON, Canada; ^6^ Department of Pediatrics, Western University, London, ON, Canada; ^7^ Mila, Quebec AI Institute, Montréal, QC, Canada

**Keywords:** cytokines, neutrophil binding, endothelial function, MAPK, ICAM-1, COVID-19

## Abstract

**Background:**

Severe COVID-19 is associated with neutrophilic inflammation and immunothrombosis. Several members of the IL-17 cytokine family have been associated with neutrophilic inflammation and activation of the endothelium. Therefore, we investigated whether these cytokines were associated with COVID-19.

**Methods:**

We investigated the association between COVID-19 and circulating plasma levels of IL-17 cytokine family members in participants to the Biobanque québécoise de la COVID-19 (BQC19), a prospective observational cohort and an independent cohort from Western University (London, Ontario). We measured the in vitro impact of IL-17F on intercellular adhesion molecule 1 (ICAM-1) cell surface expression and neutrophil adhesion on endothelial cells in culture. The contribution of two Mitogen Activated Protein Kinase (MAPK) pathways was determined using small molecule inhibitors PD184352 (a MKK1/MKK2 inhibitor) and BIRB0796 (a p38 MAPK inhibitor).

**Results:**

We found increased IL-17D and IL-17F plasma levels when comparing SARS-CoV-2-positive vs negative hospitalized participants. Moreover, increased plasma levels of IL-17D, IL-17E and IL-17F were noted when comparing severe versus mild COVID-19. IL-17F, but not IL-17A, was significantly elevated in people with COVID-19 compared to healthy controls and with more severe disease. In vitro work on endothelial cells treated with IL-17F for 24h showed an increase cell surface expression of ICAM-1 accompanied by neutrophil adhesion. The introduction of two MAPK inhibitors significantly reduced the binding of neutrophils while also reducing ICAM-1 expression at the surface level of endothelial cells, but not its intracellular expression.

**Discussion:**

Overall, these results have identified an association between two cytokines of the IL-17 family (IL-17D and IL-17F) with COVID-19 and disease severity. Considering that IL-17F stimulation promotes neutrophil adhesion to the endothelium in a MAPK-dependent manner, it is attractive to speculate that this pathway may contribute to pathogenic immunothrombosis in concert with other molecular effectors.

## Introduction

COVID-19 is an infectious disease attributable to the SARS-CoV-2 virus. Worldwide, nearly 800 million people have been infected by the virus since 2020. The wide range of symptoms in COVID-19 cases makes the disease extremely heterogeneous as some individuals have asymptomatic to mild flu-like symptoms and others can be severely ill, necessitating hospitalization in intensive care units (1). In the most severe cases, some hallmarks of the disease are neutrophilia, immunothrombosis, endothelial dysfunction (2), and acute respiratory distress syndrome (ARDS) (3–12). These severe cases also show a dysfunctional activation of the immune system that ultimately leads to organ damage (3). Many cytokines are involved in the immune response associated with COVID-19. The notable increase in interleukin 6 (IL-6) and accompanying neutrophilia raises the possible involvement of the interleukin 17 (IL-17) cytokine family, known to be associated with neutrophilia and a potent activator of the endothelium (13). Accordingly, high saliva levels of IL-17A have been associated with COVID-19 severity (14).

On the whole, IL-17 is associated with host protection against infections, and when deregulated can lead to autoimmune and inflammatory diseases. The IL-17 cytokine family consists of six isoforms, IL-17 A through F, utilizing the five IL-17 receptors, IL-17RA to IL-17RE (15). IL-17A is the most studied isoform of the family. Although IL-17F and IL-17A are highly homologous and share many physiological functions, the biological role of IL-17F remains less understood. One distinction is that IL-17A is mainly produced by T helper cells (Th17), while IL-17F is, in addition to Th17 cells, produced by innate immune cells and epithelial cells (16). Both IL-17A and IL-17F bind the IL-17RA/RC heterodimer. It is reported that IL-17A activation of the receptor is 10 to 30 times more potent than IL-17F binding, which emphasizes the differences in affinity and the vital role that IL-17A plays in driving autoimmunity (17). The activation of the IL-17RA/RC heterodimer leads to the activation of the canonical nuclear factor (NF-**κ**B) as well as the mitogen-activated protein kinase (MAPK) pathways (17). All these factors lead to the triggering of the activation of the transcription of IL-17 signature genes including cytokines and chemokines driving neutrophil recruitment (16, 17). The MAPK pathways include extracellular signal-regulated kinase (ERK), p38, and JUN N-terminal kinase (JNK) (18). A previous study showed how IL-17A promotes endothelial activation and neutrophil recruitment in a p38 MAPK-dependent matter (13).

Neutrophil adhesion is part of the five steps of the neutrophil recruitment cascade. It requires many adhesion molecules. Essentially, when endothelial cells are activated by proinflammatory cytokines, adhesion molecules such as selectins and intercellular adhesion molecule 1 (ICAM-1) have their expression increased within minutes (19). These molecules are essential for the initial steps of the recruitment cascade to capture circulating neutrophils. ICAM-1 molecules are present at the surface of endothelial cells and form a bond with a receptor at the surface of neutrophils and lymphocyte function-associated antigen 1 (LFA1). ICAM-1 binding to LFA1 is vital for firm neutrophil adhesion: the bound neutrophils are able to continue the recruitment cascade, ultimately leading to transmigration (19).

In this study, we have investigated the impact of IL-17F activation on endothelial cells and its impact on neutrophil recruitment and ICAM-1 expression at the surface level in the context of acute COVID-19.

## Methods

### Datasets

We obtained clinical data and information on circulating proteins from the Biobanque québécoise de la COVID-19 (BQC19; www.quebeccovidbiobank.ca) (20). Validation was carried out in data obtained from Western University (London, Ontario) as previously reported (21).

### Cell culture

Primary human umbilical vein endothelial cells (HUVECs) were obtained from American Type Culture Collection (ATCC) (PCS-100-013). These cells were derived from 10 individual donors, minimizing the variability associated with material derived from a single donor. The HUVECs were grown in 199 media (316-020-CL, Wisent Bio Product), supplemented with 20% FBS (080-450, Wisent Bio Product), 60 μg/ml endothelial cell growth supplement (Cedarlane, R&D system, CCM027), 2mM L-glutamine (609-065-EL, Wisent Bio Product), 50 μg/ml heparin (H4784-250MG, Sigma Aldrich), and a 1:100 dilution of penicillin-streptomycin 100X (450-201-EL, Wisent Bio Product). However, during treatments, the penicillin was removed from the growing media to avoid any interactions. The cells were cultured from passages 2-7 (13). HUVECs were plated on a gelatin-coated surface [100mm diameter Fisherbrand™ Petri Dishes (Catalog #FB085712), 6-well clear flat bottomed TC-treated plates (83,3920,005, SARSTEDT) and 24-well flat bottom with lid, TC-treated, and sterile (3526, Corning Incorporated) plates]. The sterile coating solution comprised gelatin powder 0.5% (G-1890-500G, Sigma Aldrich) in PBS (311-010-CL, Wisent Bio Product). A 2-hour minimum incubation was required for the coating at 37°C. The gelatin-coated plates could then be kept at 4°C for up to 4 weeks. The cell culture manipulations were done in a 1300 Series A2 biological safety cabinet (BSC) (ThermoFisher).

### Endothelial cells stimulation

The recombinant human IL-17F was purchased from Cerdarlane/R&D (#1335-IL-025/CF). In total, 25 μg of IL-17F was reconstituted in 250 μL of HCl 4mM to give a 100 μg/ml stock solution. The working solution used in this study was 100 ng/ml of IL-17F. The negative control consisted of fully complemented 199 media, changed on the day of the experiment. PD184352 (PZ0181-5MG, Sigma Aldrich), a selective non-competitive inhibitor of MEK (MKK1: MAPK kinase), was used at a final concentration of 2μM in DMSO to prevent ERK1/2 activation. BIRB 796 (#5989, Bio-techne), an allosteric high-affinity p38 MAPK inhibitor, was used at a final concentration of 0.1 μM in DMSO to prevent p38a/b MAPK activation. HUVECs were pre-exposed for 1 h to the inhibitors, followed by a 24-h exposure to the 100 ng/ml IL-17F treatment.

### Immunofluorescence

In the 24-well plates, 12 mm diameter round glass coverslips (Catalog# 170-C12MM, Ultident) were used. The reagents used for the cell fixation and permeabilization were PBS + paraformaldehyde (PFA) 4% (Catalog # J19943.K2, ThermoFisher), PBS + Bovine Serum Albumin 1% (BSA) (catalog #9048-46-8, Sigma Aldrich), and finally, PBS + triton 0.2% (Catalog #HFH10, ThermoFisher). Following permeabilization, a 30-min blocking step was performed using a solution of 3% BSA in PBS (catalog #A7906-50G, Sigma Aldrich). The fixed cells were then incubated for 1 h with the primary ICAM-1 antibody (catalog #MEM-111, ab2213, Abcam) at a concentration of 1:200 in the blocking solution, followed by extensive washing. They were then incubated for 30 min with DAPI (Catalog #62248, ThermoFisher) at a concentration of 1:1000 in PBS+BSA 3% to stain the DNA and visualize the nuclei. Phalloidin-TRITC (Catalog# 5783, Bio-Techne) was used to visualize the actin cytoskeleton at a concentration of 1:100 in PBS+BSA 3% and the secondary antibody that was used to stain for ICAM-1 was Alexa Fluor 488 goat anti-mouse (catalog # A11029, Invitrogen) at a 1:500 concentration. The coverslips were mounted using Fluoromount-G™ Mounting Medium (Catalog #00-4958-02, ThermoFisher). The slides were left to dry and conserved at 4°C for confocal imaging analysis.

### Neutrophil binding assay

HUVECs were grown at 90% confluency on gelatin-coated coverslips in 24-well plates. IL-17F treatment was applied to the cell for 30 min. Human peripheral neutrophils were isolated from the blood of healthy donors following this protocol (22). Once the neutrophils were isolated, they were stained using calcein-AM 1 mM stock (catalog # C1430, Invitrogen) for 30 min in RPMI (Catalog # 350-000-CL, Wisent Bio Product) at 37°C. After washes, the neutrophils stained were added to the IL-17F-treated cells for 30 min. Fixation with PFA 4% and staining were as described for the immunofluorescence analysis.

### Imaging analysis

The imaging was done using a Zeiss LMS700 confocal microscope and a Zeiss Axio Imager M2. A 20x objective was used to obtain the results. All of the analysis of the images was done using Fiji/ImageJ software for Microsoft Windows.

### Flow cytometry

Acquisition of the flow cytometry samples was done using a BD FACSCanto II system. After 24 h of IL-17F treatment, cells were detached from the 6-well plates with trypsin (Catalog #325-045-EL, Wisent Bio Product). The cells were transferred into FACS tubes (catalog# 149595, Fisher Scientific) for flow staining. The viability marker was incubated for 20 min on ice (Catalog #423102, BioLegend). An Fc receptor-blocking solution was then applied for 10 min on ice (catalog #422302, Biolegend). Fixing/permeabilization of the samples was done using the BD Cytofix/CytoPerm and the BD Perm/Wash kits (catalog #554772/554723, BD). The primary antibody ICAM-1 1:1000 and secondary antibody Alexa Fluor 488 1:1000 were incubated for 30 min. The samples were fixed using 2% PFA and filtered before flow acquisition. The software FlowJo 10.2 was used to analyze the experiment.

### Statistical analysis

The data were analyzed on the GraphPad software version 9.2. The Shapiro-Wilk normality test was done on all data to ensure normality and allowed us to use parametric tests such as one-way ANOVA followed by Tukey’s multiple comparisons test.

## Results

### IL-17F, but not IL-17A, is elevated in severe COVID-19

**Figure 1:**
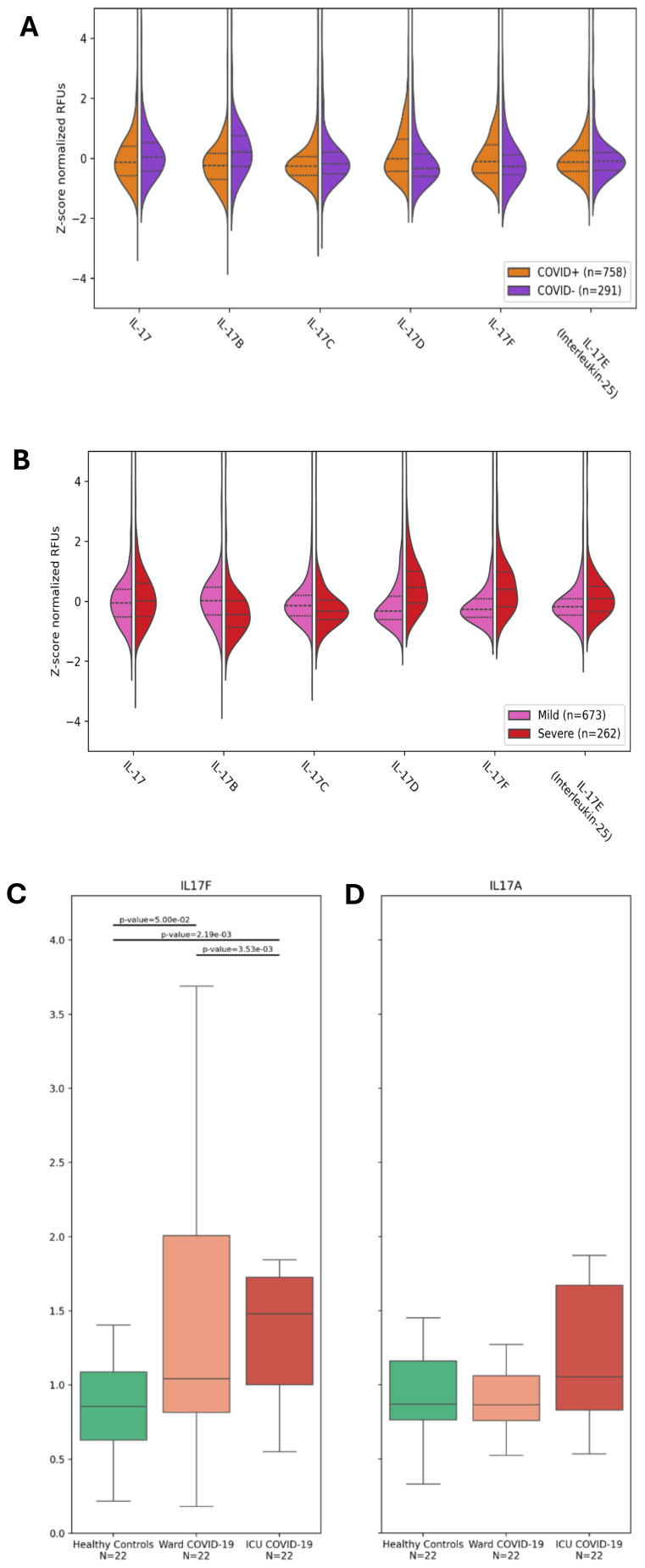
IL-17F is enriched in severe COVID-19 but not IL-17A. **(A, B)** Violin plots showing the enrichment or depletion of the IL-17 cytokine family. **(A)** Shows infected patients (n=758) vs uninfected patients (n=291). **(B)** Shows disease severity, comparing mild (n=673) and severe (n=262) conditions. The y-axis shows the z-score normalization of the relative fluorescence units (RFUs). These values were obtained from the plasma of BQC19 participants, seeking to measure circulating levels of the IL-17 family (on the x-axis). Data were analyzed using the two-tailed Wilcoxon rank-sum test. **(C, D)**. IL-17F **(C)** and IL-17A **(D)** levels were measured in the plasma of healthy controls (n=22) and COVID-19-participants from the ward (n=22) or the ICU (n=22) using an Olink Explore 1196 panel as described previously (21). The bar graphs represent the normalized values (NPX) for each of the two cytokines. Data were analyzed using the two-sided Mann-Whitney U test. The bars represent the compared groups, with the exact *p* values reported. The absence of bars means the comparison was not significant.

As aforementioned, severe COVID-19 is associated with some hallmark conditions such as neutrophilia, immunothrombosis, and ARDS (3–12). Since the IL-17 cytokine family, and in particular, IL-17A and IL-17F, has been associated with neutrophilic inflammation (4, 8, 9, 23, 24), we investigated the abundance of IL-17 cytokines in the plasma of hospitalized participants of the BQC19, a prospective observational cohort (20), using data from a SomaScan 5K array, an aptamer-based multiplex technology (25). We first compared the plasma levels of the IL-17 cytokines between SARS-CoV-2-positive (*n*=758) and negative (*n*=291) participants. No differences were observed for IL-17E (IL-25), a cytokine associated with Th2 responses rather than Th17 (**Table 1** and **Figure 1A**). We found decreased levels of IL-17A, IL-17B, and IL-17C and increased levels of IL-17D and IL-17F in the SARS-CoV-2 positive hospitalized participants compared with the negative hospitalized participants, with the most significant changes being for IL-17B and IL-17D (**Table 1** and **Figure 1A**). We then investigated whether IL-17 cytokines were associated with disease severity using the WHO classification (1), comparing only SARS-CoV-2-positive hospitalized participants in the BQC19 cohort (mild: *n*= 262; severe: *n*= 673). No significant differences were observed for IL-17A, but we found decreased levels of IL-17B and IL-17C and increased levels of IL-17D, IL-17E, and IL-17F in patients with severe COVID-19 compared with patients with mild COVID-19, with the most significant changes being for IL-17D and IL-17F (**Table 1** and **Figure 1B**). To confirm the findings obtained from participants in the BQC19, we validated the results in an independent cohort from Western University (London, Ontario) (21). Analysis of the circulating proteins in these samples was performed on a different platform, an Olink Explore 1196 panel (21). In accordance with the findings obtained in BQC19, IL-17F levels were significantly elevated in COVID-19-positive participants from the ward (p=0.05) or ICU (p=0.0022) compared to the healthy controls (**Figure 1C**). Moreover, the COVID-19-positive participants from the ICU had significantly greater levels than those from the ward (p=0.0035) (**Figure 1C**). In contrast, IL-17A levels were not significantly elevated in the COVID-19-positive participants from the ward or ICU compared to the healthy controls (**Figure 1D**). While IL-17A and IL-17F signal through the same receptor complex (IL-17RA/RC) and have been proposed to act redundantly (17), the results obtained from investigating these two cohorts show that IL-17F but not IL-17A was associated with SARS-CoV-2 infection in the hospitalized participants and that IL-17F but not IL-17A levels are associated with more severe disease. Due to the nature of the two proteomic analyses, we can only report the relative values of the cytokines, which is a limitation when assessing such a large number of proteins simultaneously.

**Table 1:** IL-17 family target enrichment.

Cytokine	Analysis	Enriched or depleted	p-value	Corrected p value
**IL-17 A**	COVID+ vs COVID-	depleted	0.008126	0.012188832
**IL-17 B**	COVID+ vs COVID-	depleted	1.76E-18	1.05E-17
**IL-17 C**	COVID+ vs COVID-	depleted	0.018907	0.022688623
**IL-17 D**	COVID+ vs COVID-	enriched	3.88E-09	1.17E-08
**IL-17 F**	COVID+ vs COVID-	enriched	0.000387	0.00077347
**IL-17 E**	COVID+ vs COVID-	depleted	0.651637	0.651637108
**IL-17 A**	Mild vs Severe COVID	enriched	0.059217	0.059216624
**IL-17 B**	Mild vs Severe COVID	depleted	1.08E-15	2.16E-15
**IL-17 C**	Mild vs Severe COVID	depleted	6.18E-06	7.42E-06
**IL-17 D**	Mild vs Severe COVID	enriched	2.92E-32	1.75E-31
**IL-17 F**	Mild vs Severe COVID	enriched	4.80E-28	1.44E-27
**IL-17 E**	Mild vs Severe COVID	enriched	5.10E-10	7.65E-10

### IL-17F drives ERK1/2 and p38 MAPK-dependent neutrophil adhesion on HUVECs

**Figure 2:**
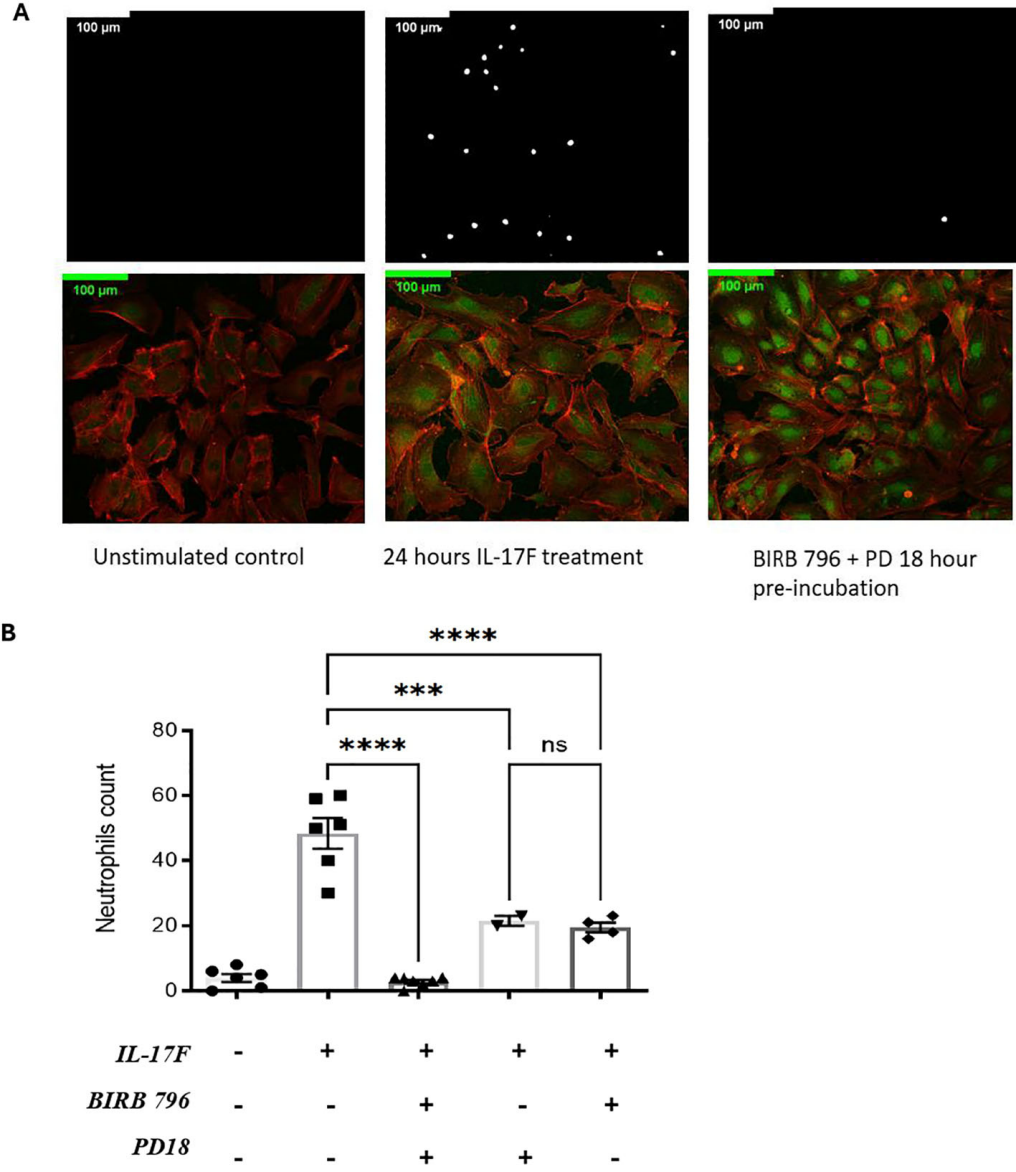
IL-17F drives ERK1/2 and p38 MAPK-dependent ICAM-1 cell surface expression and adhesion of neutrophils in HUVECs. **(A)** Neutrophils were incubated with calcein-AM for 24 h. After incubation, endothelial cells were treated with IL-17F for 24 h and BIRD 796 + PD18 for 1 h. The white point represents neutrophils binding to treated endothelial cells (top panels). Immunofluorescence of HUVECs under different conditions are shown (bottom panels). F-actin was stained with phalloidine 565 (red) and ICAM-1 with Alexa 488 indirect staining (green). Cells were visualized using a Zeiss LMS700 confocal microscope and a Zeiss Axio Imager M2. Fiji/Image J was used to analyze the images. Representative images are shown at ×20 magnification (n = 4). **(B)** Representative of the neutrophil counts under different conditions (n=2-). Data are presented as mean ± SEM and were compared with a one-way ANOVA followed by Tukey’s multiple comparisons test with a pair-wise comparison between the positive control (Il-17F+/BIRB 796-/PD18-) and the three other groups. Significance levels are shown as ns (non-significant), ***(p<0.001), and **** (p<0.0001). HUVEC, human umbilical vein endothelial cell.

We previously showed that an important target of IL-17A is the endothelium regulating neutrophil adhesion *in vivo* (13). Considering the results demonstrating that in severe COVID-19, IL-17F levels are increased, we investigated whether IL-17F alone was sufficient to activate endothelial cells *in vitro* and mediate neutrophil adhesion. HUVECs, a well-established model, were exposed to IL-17F for 24 h and then incubated with freshly isolated calcein-labeled human neutrophils for 30 min before washing the unbound cells and enumerating the adhered neutrophils. The HUVECs were simultaneously stained for ICAM-1 to visualize its expression using immunofluorescence. We observed that ICAM-1 expression was low in the untreated HUVECs (**Figure 2A**). This was associated with very few neutrophils adhering to the surface of endothelial cells (4 ± 1.2) (**Figures 2A, B**). When treated with 100 ng/ml of IL-17F for 24 h, the expression of ICAM-1 was increased (**Figure 2A**) and >10 times more neutrophils were able to adhere (48 ± 4) (**Figures 2A, B**). We previously showed that p38 MAPK inhibition prevented ICAM-1 expression and neutrophil adhesion in response to IL-17A (13). Preincubating HUVECs with either BIRB0796 to block p38 MAPK activity or PD184352 to prevent ERK1/2 activation partially decreased the number of neutrophils adhering in response to IL17F (one-way ANOVA, F= 55, p<0001; followed by Tukey’s multiple comparisons test: 21 ± 1.5 ***, p = 0.0003 and 19.5 ± 1.6 ****, p=0.0001). However, when combining the two MAPK pathway inhibitors, PD184352 and BIRB 796, the adhesion of neutrophils was reduced to levels comparable to untreated cells (3 ± 0.5 vs. 48 ± 4 ****, p= <0.0001) (**Figure 2B**). The results suggest that both p38 MAPK and ERK1/2 contribute to the adhesion of neutrophils to endothelial cells mediated by IL-17F.

Surprisingly, when examining ICAM-1 expression using immunofluorescence, the results revealed that the MAPK pathway inhibitors did not inhibit ICAM-1 protein expression (**Figure 2A** bottom panel). Closer examination revealed that while the intensity of the signals was not decreased, its localization may have been affected, with more pronounced perinuclear staining in the presence of BIRB0796 and PD184352. Indeed, as mentioned previously, ICAM-1 is one of the essential molecules for the interaction and the adhesion of neutrophils on the surface of endothelial cells. If ICAM-1 is still being expressed but not at the right place, the neutrophils may not be able to interact with the HUVECs, thereby reducing the number of adhered neutrophils as observed.

### An ERK1/2 and p38 MAPK-dependent increase in ICAM-1 cell surface expression in IL-17F-treated HUVECs

**Figure 3:**
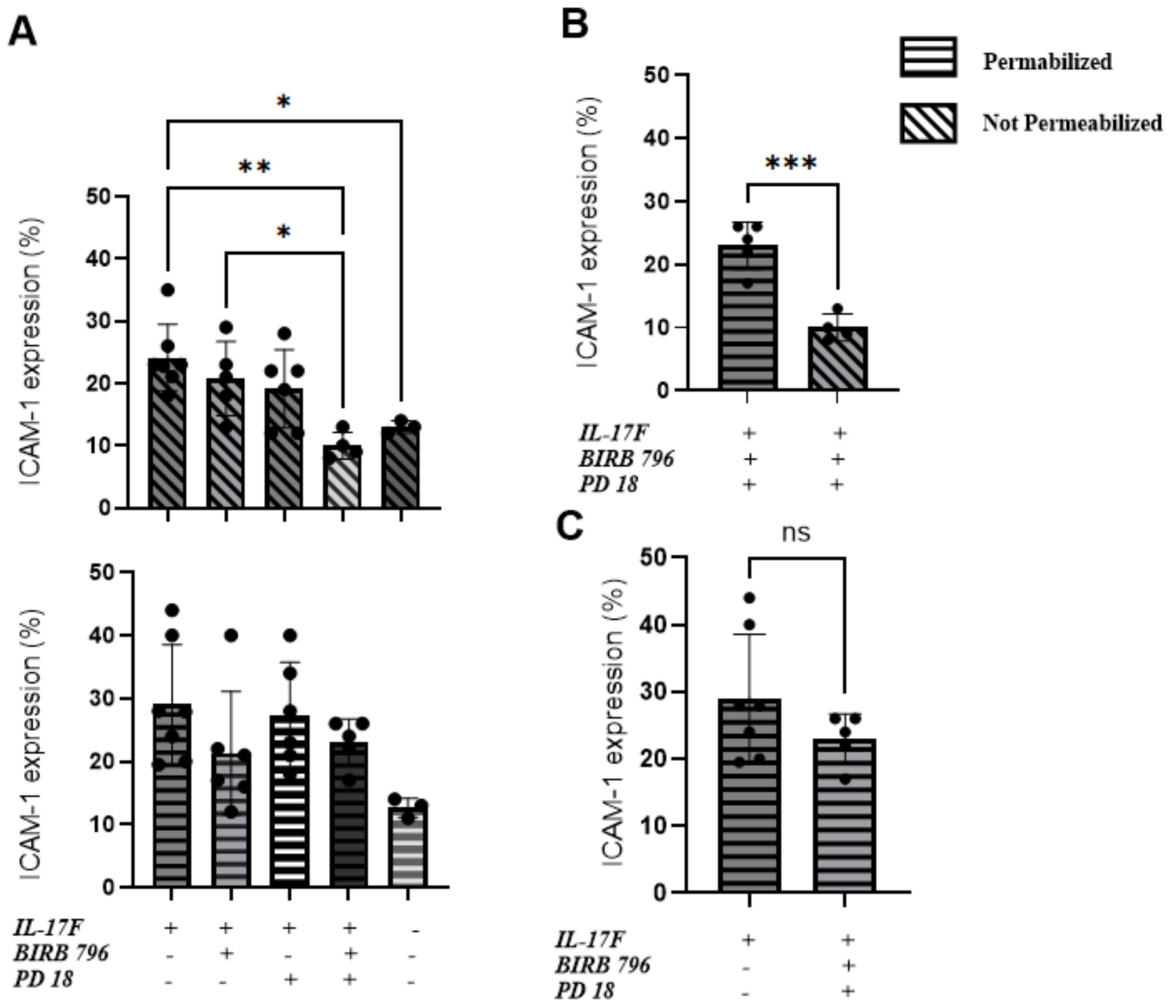
An ERK1/2 and p38 MAPK-dependent increase in ICAM-1 cell surface expression in IL-17F-treated HUVECs. Percentages of total ICAM-1 expression compared to cell surface expression (n=5). This represents the percentage of ICAM-1-positive cells based on our flow cytometry gating strategy accessible on page 9 of the raw data submission. Data are presented as mean ± SEM Statistical significance was shown using a one-way ANOVA followed by Tukey’s multiple comparisons test. **(A)** The top panel is the not-permeabilized group in the experiment whereas the bottom panel is the permeabilized group in the experiment. **(B)** Comparison between the permeabilized and not permeabilized groups in the groups with the two inhibitors. **(C)** The IL-17F group vs the group with the two inhibitors, showing no statistically significant difference in ICAM-1=positive cells. The permeabilized group is represented by horizontal hatched lines and the not permeabilized group is represented by diagonal hatched lines. The x-axis shows the percentage of cells positive for ICAM-1 Significance levels are shown as ns (non-significant), *(p<0.05), **(p<0.01), and ***(p<0.001). HUVEC, human umbilical vein endothelial cell.

We conducted flow cytometry analyses to compare the total expression of ICAM-1 with the surface-level expression. The goal was to determine whether the MAPK pathway inhibitors affected ICAM-1 protein expression at the surface of HUVECs following 24 h of IL-17F treatment. The cells were permeabilized to measure the total ICAM-1 expression or left unpermeabilized, allowing the ICAM-1 antibody to only bind to the ICAM-1 protein at the surface of the cells. The basal level of ICAM-1 expression was 13% ± 1.5% for both the permeabilized and not permeabilized conditions. In the not permeabilized group only (**Figure 3A** top panel) our one-way ANOVA followed by Tukey’s multiple comparisons test was able to show significance when comparing the IL-17F-only group with the group with both inhibitors (**p=0.0023). There was also significance between the basal level and the IL-17F group (p=0.04) and finally between the group with both inhibitors and the BIRB 796 group (p=0.04) The combination of both inhibitors showed a significant reduction in ICAM-1 expression at the surface level (**Figure 3B**) (23.0% ± 1.7% vs 10.0% ± 1.1%***, p = 0.0005). These findings are coherent with the neutrophil adhesion assay, where the presence of both the MAPK inhibitors restored a basal-level phenotype (i.e., no neutrophil adhesion and basal-level ICAM-1 surface expression). This suggests that to inhibit neutrophil adhesion to similar basal level, the inhibition of both the MAPK pathways (p38 MAPK and ERK1/2) is required. In agreement with our immunofluorescence findings, the presence of the MAPK pathway inhibitors did not significantly reduce the total expression of ICAM-1 in response to IL-17F (p= 0.2, ns) (**Figure 3C**). This suggests that the ICAM-1 protein is still being expressed but not, however, at the surface level for the interaction with the neutrophils, preventing their adhesion.

## Discussion

Overall, our analysis of the plasma from participants to BQC19 revealed increased levels of IL-17F and IL-17D when comparing SARS-CoV-2-positive and SARS-CoV-2-negative hospitalized participants. Interestingly, IL-17A levels were decreased in this comparison, but it is important to note that the COVID-19-negative participants are not healthy controls, but participants who were hospitalized for suspected COVID-19 but were found to be negative for SARS-CoV-2. The underlying condition leading to their hospitalization may well have led to a significant increase in IL-17A that is greater than that elicited by SARS-CoV-2. Another notable finding was the cytokines associated with disease severity. The levels of IL-17F and IL-17D were found to be significantly elevated, while IL-17A showed no significant difference when comparing severe SARS-CoV-2 and mild SARS-CoV-2 hospitalized participants. Although Il-17A and IL-17F are known to signal through the same receptor IL-17RA/RC, the results obtained here from the analysis indicate that IL-17F and not IL-17A was significantly enriched in the plasma of patients with severe COVID-19. We confirmed this difference between IL-17F and IL-17A in an independent cohort from Western University (21), where similar observations were made (**Figures 1C, D**). Interestingly, not only is this cohort independent, but the study also used a different technology to assess the protein levels, further strengthening the conclusion that the observed changes reflect the cytokine levels and not the method of detection. This does not mean that IL-17A is absent in severe COVID-19 or that it does not play a role in the pathobiology of the disease. Previous studies showed an association of IL-17A salivary levels with COVID-19 severity (14), as well as an imbalance in the Th17/Treg axis (26), leading to the suggestion that an IL-17A blockade could constitute a viable therapeutic strategy (27, 28). However, the BISHOP study, which investigated the blockade of IL-17A with secukinumab in hospitalized COVID-19 patients, failed to show efficacy in treating COVID-19 (29). Our results show an association between IL-17F and COVID-19 severity in the large BQC19 cohort rather than IL-17A, which may provide an explanation for the lack of efficacy when targeting IL-17A alone. The results also suggest that either targeting IL-17F or abrogating the activity of both cytokines, targeting their common downstream receptor IL-17RC, could be another therapeutic avenue to attenuate the severe outcomes of COVID-19.

**Figure 4:**
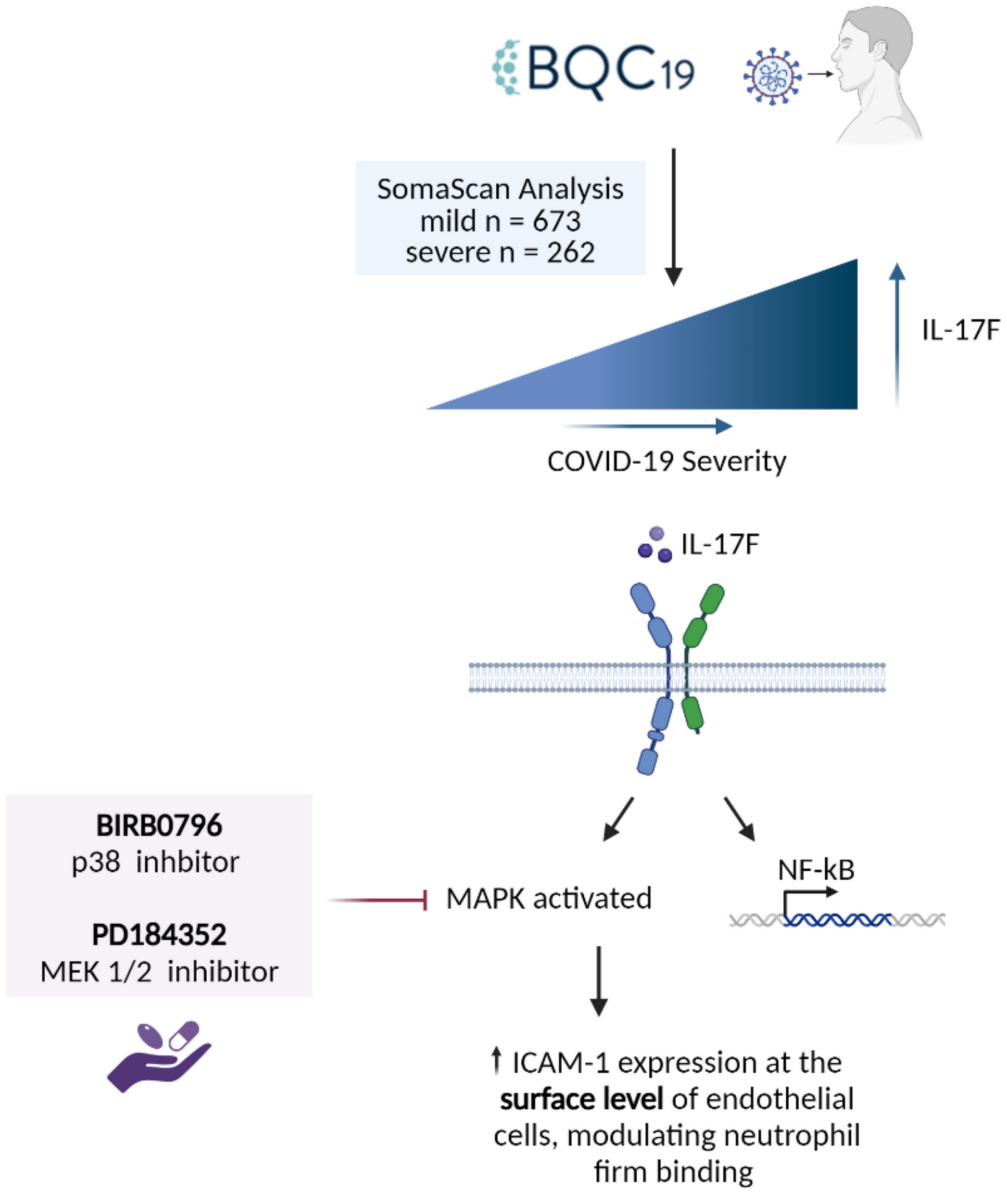
Summary of the findings. IL-17F was found to be significantly enriched in the patients with more severe forms of COVID-19. IL-17F activates the IL-17RA/RC receptor which leads to the activation of MAPK pathways as well as transcription factors such as NF-κB. The activation of the MAPK pathways (p38 and ERK) leads to the increase of ICAM-1 expression at the surface level of endothelial cells, thereby increasing neutrophil adhesion. When two MAPK inhibitors are introduced, neutrophil adhesion is significantly reduced as well as the expression of ICAM-1 at the surface level.

In the cell-based experiments, we focused initially on IL-17F given its close relationship with IL-17A in terms of downstream signaling. Following the activation of IL-17 receptors, the MAPK and NF-**κ**B pathways are activated (30, 31), which regulates downstream effector function via the upregulation of proinflammatory molecules such as chemokines, cytokines, adhesion molecules, and matrix metalloproteinases (32). All these molecules play important roles in the proinflammatory state of endothelial cells and the recruitment of immune cells, such as neutrophils, to the site of infections (16). They can contribute to hallmark features of severe COVID-19 disease such as neutrophilia, immunothrombosis, endothelial dysfunction, and ARDS (2, 7–9, 11). In this study, we focused on ICAM-1, an important adhesion molecule expressed on endothelial cells that is essential for neutrophil adhesion. ICAM-1 is a 90 kDa glycoprotein located at the cell surface. It is a ligand for the integrins present at the surface level of neutrophils, i.e., LFA1. Its activity is vital for regulating leukocyte recruitment in circulation to the sites of inflammation (33). Its soluble form has been found to be elevated in COVID-19 and is associated with angiogenesis in post-COVID conditions (34). Our results demonstrate that IL-17F activates the endothelium, increasing ICAM-1 cell surface expression and, subsequently, neutrophil adhesion. The introduction of two inhibitors of the MAPK pathways, PD184352 (an MKK1/MKK2 inhibitor) and BIRB0796 (a p38 MAPK inhibitor), led to decreased neutrophil adhesion on the surface of HUVECs (**Figure 4**). This decreased neutrophil binding was seemingly due to a reduction in the expression of ICAM-1 molecules at the surface level. ICAM-1 expression is closely associated with the activation of NF-**κ**B by cytokines such as TNFα, IL-6, IL-1β, and IL-17A (13, 33, 35). Upon joint inhibition of the p38 and ERK1/2 MAPK pathways, we observed neutrophil binding returning to the basal level but, intriguingly, ICAM-1 was still present but not expressed at the cell surface. This suggests that p38 and ERK1/2 MAPK are not required for IL-17F-driven ICAM-1 transcription in HUVECs, a role likely accomplished by NF-**κ**B. They are, however, required for cell surface expression.

IL-17A and IL-17F are known to play a role in the development of inflammation and host defense following the activation of their IL-17RA/RC receptor, leading to the expression of proinflammatory molecules (36). However, IL-17D is a considerably less studied member of the IL-17 family. Still, we found it to be elevated in SARS-CoV-2-positive participants in the BQC19 cohort and it was also associated with disease severity to an even greater extent than IL-17F. Interestingly, our findings of elevated IL-17D in COVID-19 are in accordance with, and validate, the results reported in the Western University cohort (21). IL-17A and IL-17F have approximately 50% homology, while IL-17F and IL-17D are reported to have 25% homology (36). Unlike other IL-17 family members, IL-17D has an extended C-terminal domain capable of mediating its distinctive receptor interactions (36). Until a few years ago, the IL-17D receptor was still unknown. It was recently published that IL-17D does not bind to any IL-17 receptors but rather on CD93, a glycoprotein expressed on innate lymphoid cells type 3 (ILC3) that is implicated in the production of cytokines associated with T helper cells 17 (37). In that study, mice lacking CD93 receptors on ILC3 cells had impaired IL-22 production and severe colonic inflammation. In another study, IL-17D stimulation of HUVECs increased IL-8 production in an NF-**κ**B dependent manner (38). These levels of IL-8 were physiologically relevant to inhibit hemopoiesis (36). Although knowledge of the role of IL-17D in humans is limited, IL-17D may play a role in regulating the hematopoietic response to inflammation through the production of proinflammatory cytokines derived from ILC3 (36). Another study found that IL-17D played a crucial role during intracellular bacteria and influenza A infection by suppressing the activity of CD8 T cells through the regulation of dendritic cells (39). Additional research is required to better understand the role of IL-17D in inflammation within the context of viral respiratory infections.

## Conclusion

Circulating IL-17F is associated with more severe COVID-19. Furthermore, IL-17F promotes neutrophil adhesion to endothelial cells, an event associated with ICAM-1 cell surface expression in a MAPK-dependent manner.

## Data Availability

The datasets presented in this study can be found in online repositories. The names of the repository/repositories and accession number(s) can be found in the article/**Supplementary Material**.
