# Supplementary material for: Circulating IL-17F, but not IL-17A, is elevated in severe COVID-19 and leads to an ERK1/2 and p38 MAPK-dependent increase in ICAM-1 cell surface expression and neutrophil adhesion on endothelial cells
Source: Front Immunol. 2024 Oct 18;15:1452788. doi: 10.3389/fimmu.2024.1452788 (PMC11527637; doi:10.3389/fimmu.2024.1452788)
Supplement: Supplementary file 1 [file DataSheet1.pdf]

Raw data submission

Circulating IL-17F, but not IL-17A, is elevated in severe COVID-19 and leads to ERK1/2 and p38 MAPK-dependent increase ICAM-1 cell surface expression and neutrophil adhesion on endothelial cells

Jérôme Bédard-Matteau[1,2,3], Antoine Soulé[4], Katelyn Yixiu Liu[1,2], Lyvia Fourcade[1,2], Amin Emad[4,7] Douglas D. Fraser[5,6] and Simon Rousseau[1,2,3*]

1The Meakins-Christie Laboratories at the Research Institute of the McGill University Heath Centre

Research Institute , 2Department of Medicine, Faculty of Medicine, McGill University, Montréal,

QC, Canada, 3Department of Pharmacology and Therapeutics, McGill University, Montre´ al, QC, Canada,

4Department of Electrical and Computer Engineering, McGill University, Montreal, QC, Canada,

5Children's Health Research Institute & Lawson Health Research Institute, London, ON, Canada,

6Department of Pediatrics, Western University, London, ON, Canada, 7Mila, Quebec AI Institute,

Montre´ al, QC, Canada

***** address correspondence to:

Simon Rousseau, RI-MUHC, E M3.2244, 1001 Décarie, Montréal H4A 3J1, Canada,

Email: simon.rousseau@mcgill.ca

Keywords: Cytokines, Neutrophil binding, Endothelial function, MAPK, COVID-19, ICAM-1

Content table:

- Figure 2A top panel...3
- Figure 2A bottom panel..6
- Figure 3 ICAM-1 expression gating strategy...............................9
  - Figure 2B Neutrophils adhesion numbers 9
- Figure 4 License and publication rights...................................10

Immunofluorescence Images (Figure 2A)

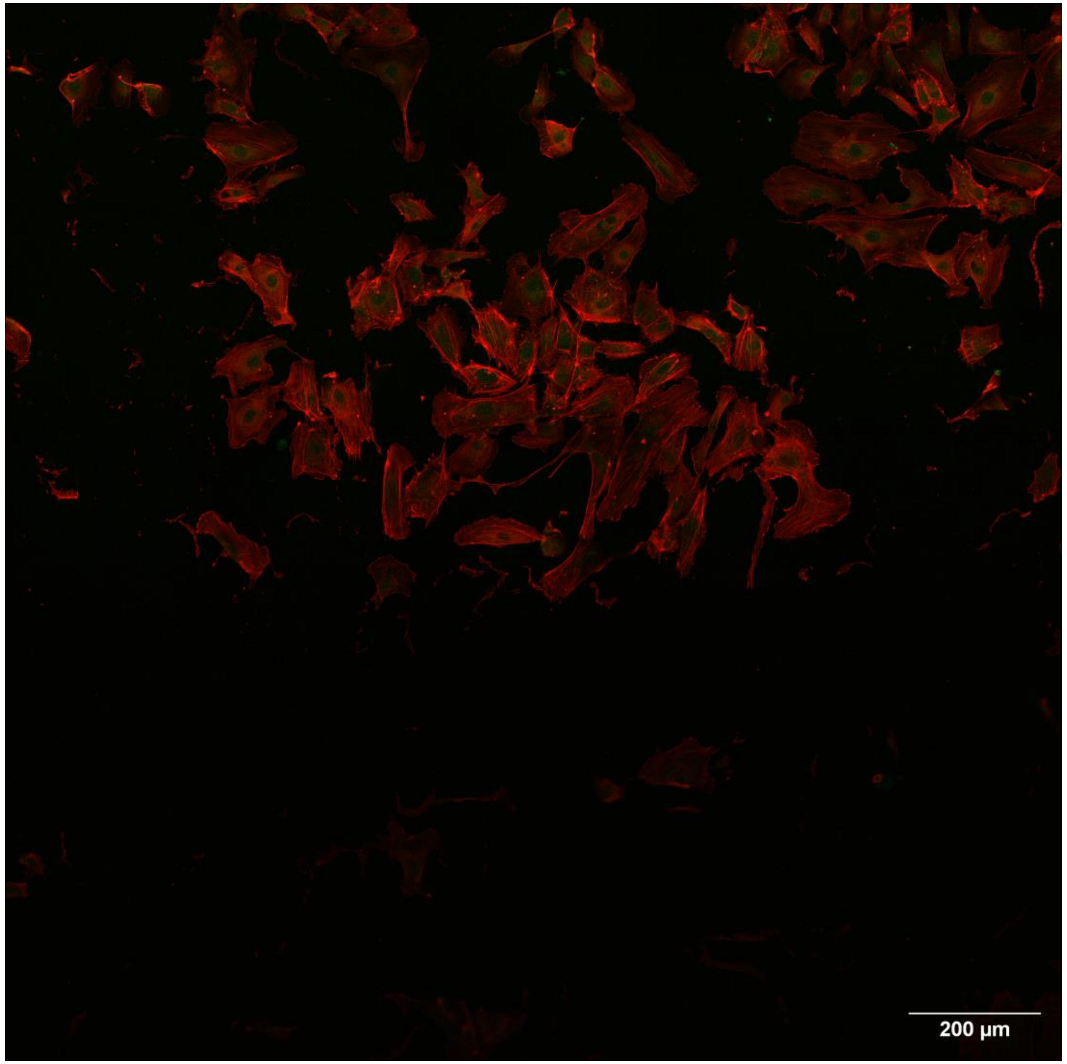

- Unstimulated control after 24 hours of Il-17F treatment. Used for figure 2A
- Red= actin, green ICAM-1

Immunofluorescence images (Figure 2A)

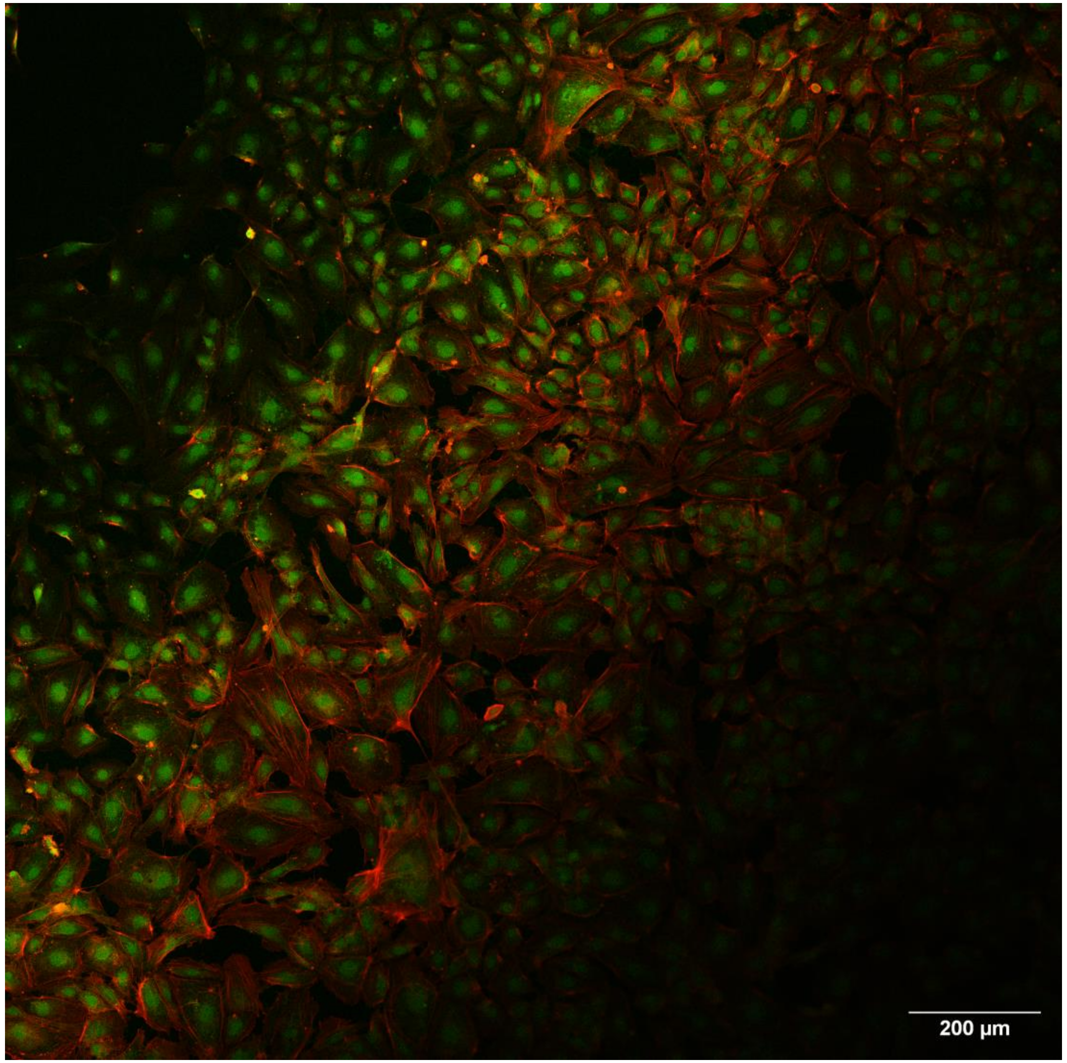

- Both inhibitor followed by IL-17F treatment 24 hours, Used in Figure 2A
- Red= actin / Green = ICAM-1

Immunofluorescence Images (Figure 2A)

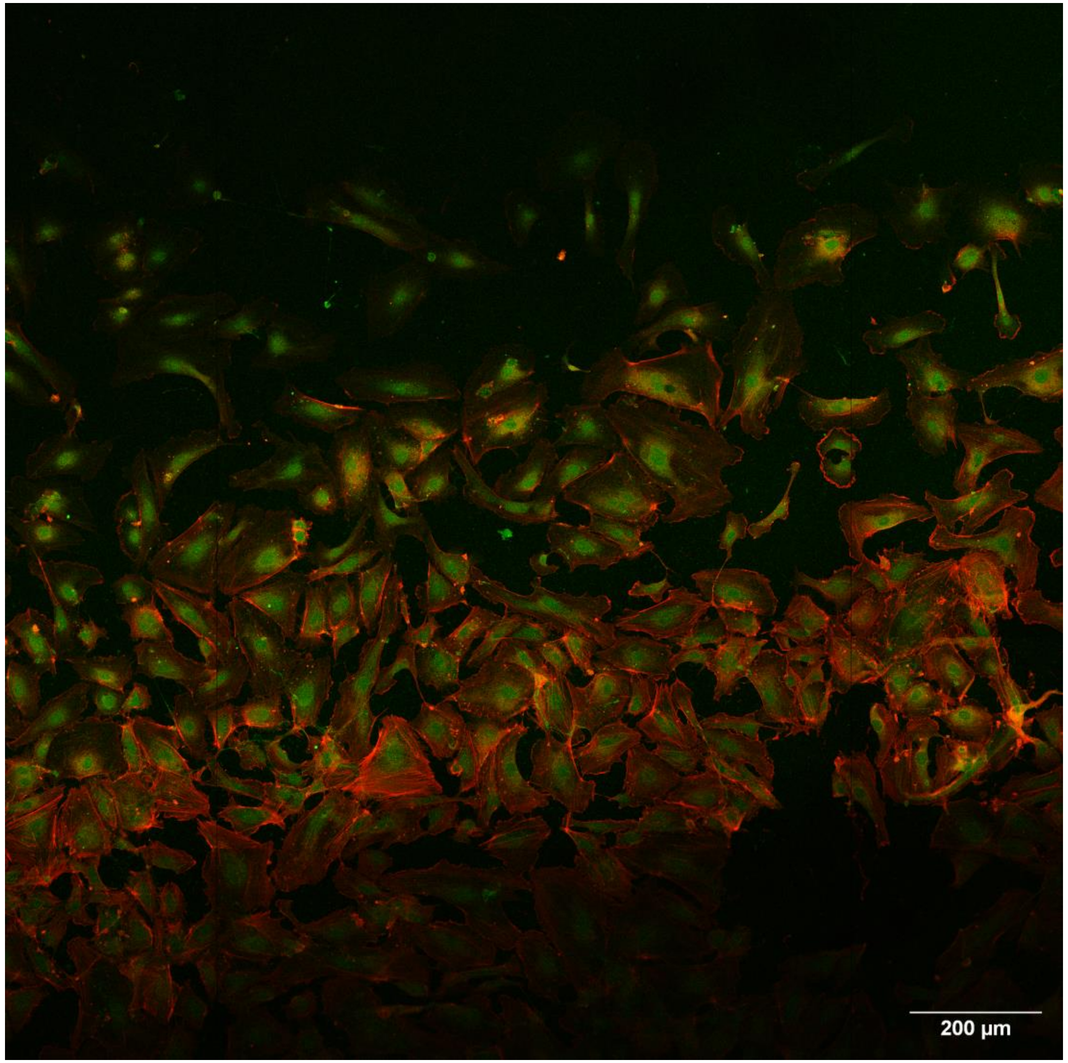

- IL-17F treatment only for 24 hours, used in figure 2A
- Red = actin / Green = ICAM-1

Neutrophils adhesion (Figure 2A)

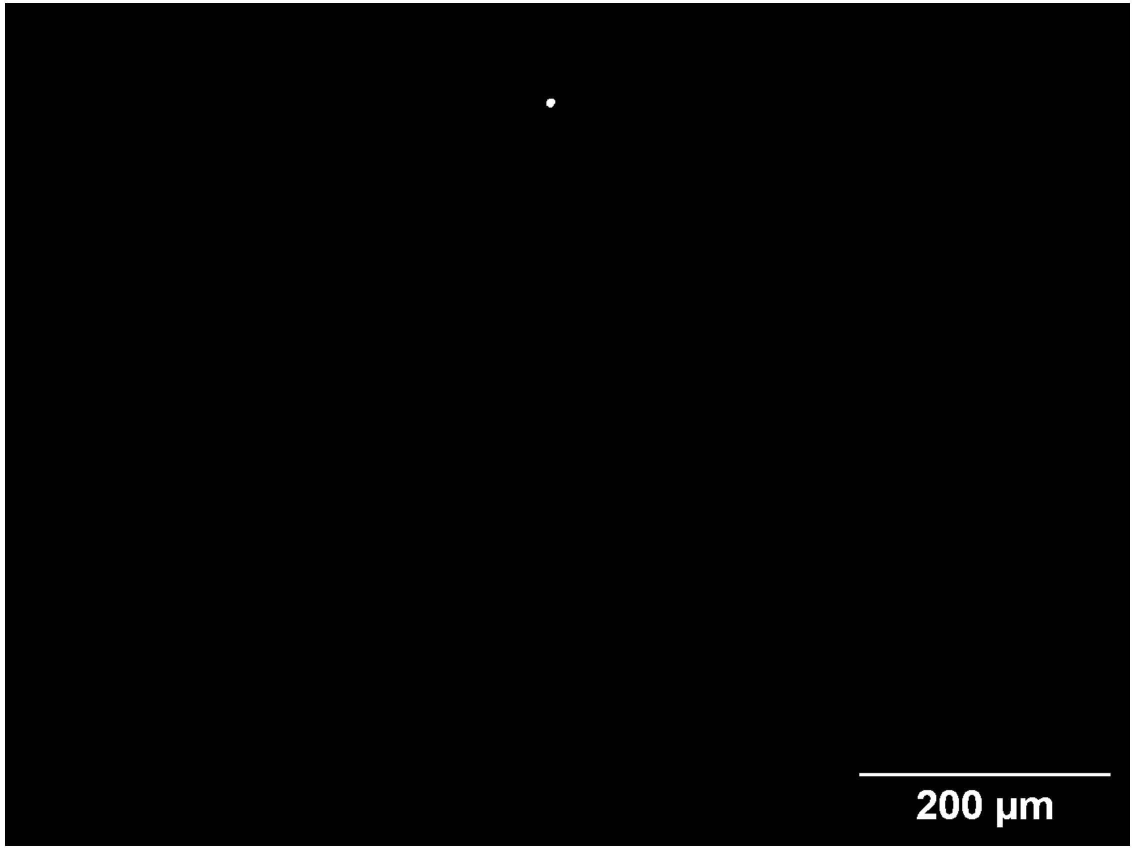

- Unstimulated control, showing neutrophils as white dots. Used for figure 2A

Neutrophils adhesion (Figure 2A)

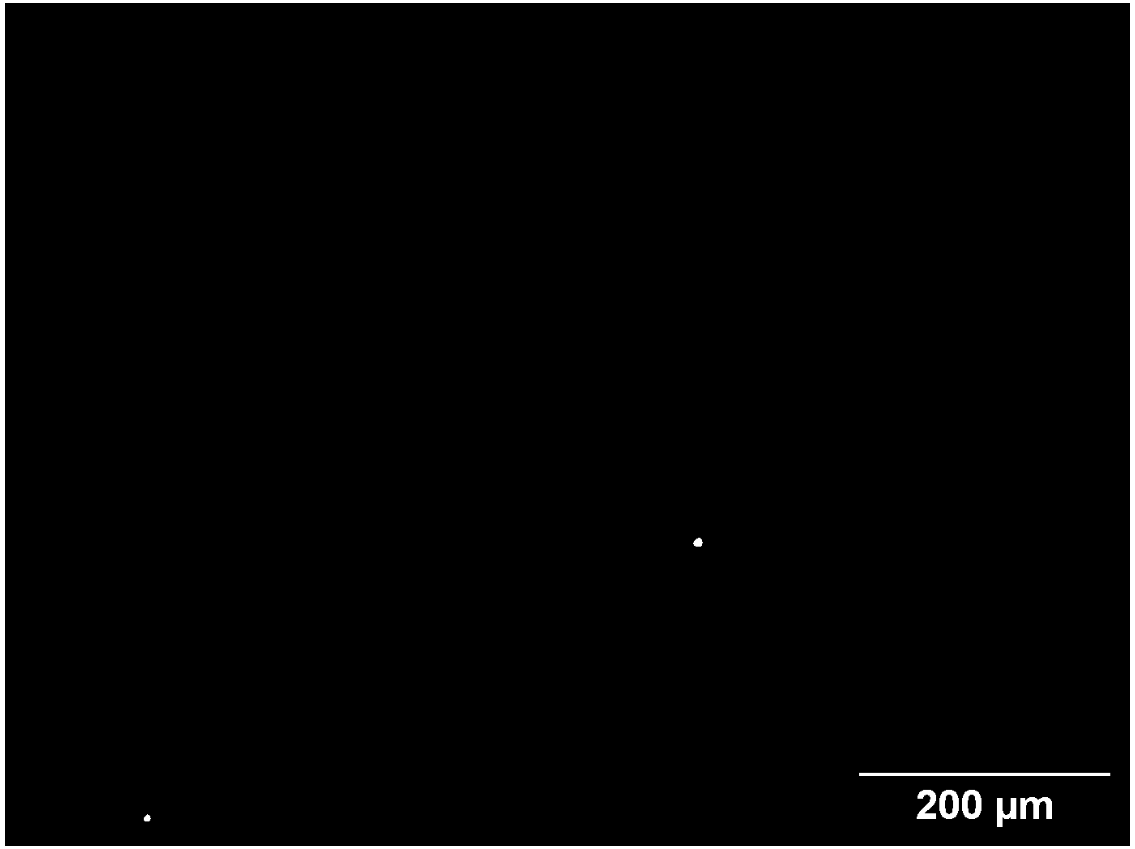

- Both inhibitors in combination for 1 hr followed by 24 hours of Il-17F treatment. The white dots represent neutrophils adhering to the surface of the endothelial cells.
- Used for figure 2A

Neutrophils adhesion (Figure 2A)

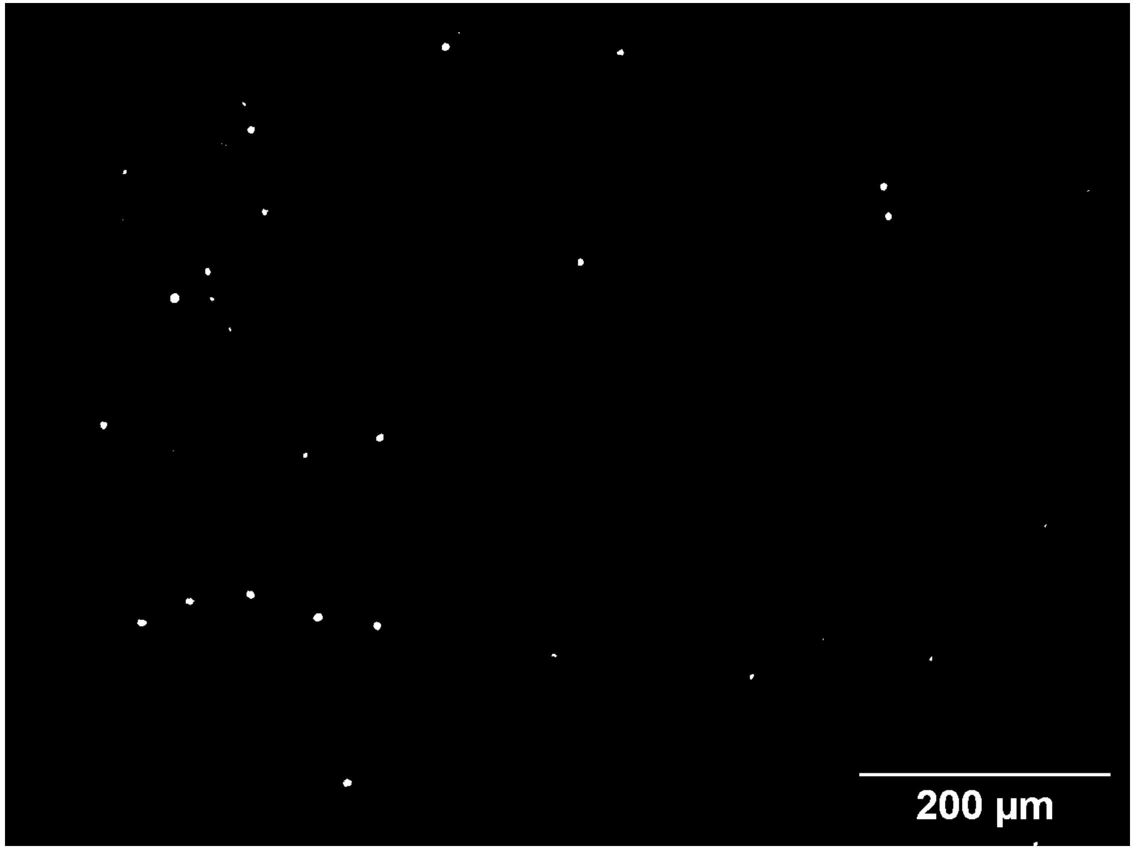

- IL-17F treatment for 24hrs. The white dots represent neutrophils binding to the surface of endothelial cells.
- Used for figure 2A
